# Allegheny County Medical Society

**Published:** 1889-09

**Authors:** 


### ALLEGHENY COUNTY MEDICAL SOCIETY.

---

SPECIAL MEETING JUNE 18, 1889.

---

Wm. F. Knox, M. D., President, in the chair.

Dr. Macfarlane reported a case of

### FATAL ALCOHOLIC POISONING IN A CHILD.

A boy, aged five years, was in good health playing on the street, when his mother called him in for dinner. A few minutes elapsed from the time the child was called until the mother was prepared to place him at the table, not over twenty minutes. When she looked around for the child, she saw that he appeared to be intoxicated. It the sitting room there was a bottle containing some six or eight ounces of whiskey, along with some cake —a visitor had come in and had been treated by the mother.

She found the bottle empty. This occurred between twelve and twenty minutes after twelve. The child was put to bed to sleep the liquor off. After the child had taken the whiskey, it became blanched, and this was followed, in an hour or so, by purging and vomiting. About six o'clock I saw the child. It had a rapidly running pulse and a normal temperature, The pupils of the eyes were dilated, and did not respond to light. There was a slight twitching about the face, and an occasional tremor about the hands and fingers. I put my nose to the child's breath and it was identical with that of a man who has been intoxicated by whiskey. I did what I could for the patient under the circumstances. I gave it bromide of potash and coffee. I saw it again at eleven o'clock, and found it in general convulsions. It died about half-past eleven, eleven hours from the time it took the liquor.

Dr. Jones reports several cases of

SECOND ATTACKS OF SEMEL-INCIDENT DISEASES.

I have recently had, under my care, a child with his second attack of scarlet fever. I had treated this child a couple of years ago, with the same disease ; and recently another child died with diphtheria, whom I had treated the second time for scarlet fever. I also treated this child the first time. I had two cases of measles in two children in one family, both of whom had two attacks of that disease within six weeks ; they got over the first attack and were up and about and almost well, seemingly, when they were again taken down with the disease.

PELVIC PERITONITIS CAUSED BY VIOLENT PRESSURE ON THE UTERUS POST-PARTUM.

DR. MUNN.—The case I relate this evening illustrates the probable and possibly frequent result of the injudicious use of Crede's method of completing the third stage of labor, and the injury that meddlesome midwifery is apt to inflict. I recently visited a woman in labor, for the purpose of removing a retained placenta. The women in attendance had made traction with the cord, and partially detached the placenta; the woman was bleeding freely. Alarmed at her condition, I immediately placed my hands above the fundus of the uterus, and exercised what I thought was a proper amount of compression—what I supposed was the exact method of Crede. The hemorrhage was controlled promptly. I continued the pressure, and a moment later the hard ball, which could be felt in my hands, disappeared. Examining through the vagina, I felt a rounded tumor in the posterior cul de sac. I had exercised too great violence, and retroflexed the uterus. The patient progressed favorably. A week later, she was seized with all the symptoms of peritonitis. For two or three weeks her condition was so serious that I daily expected her death. After that she made a slow recovery, For several months she came to my office for treatment on account of pelvic pain. I could discover nothing but an erosion of the cervix, and this I treated by the application of iodine and solutions of nitrate of silver. The thickening which resulted from the peritonitis was so marked that I could necessarily make no positive diagnosis. Under general tonic treatment she regained a fair degree of health, but was never able to attend to her household duties and still suffered from pelvic pain. She passed out of my hands. In the spring of this year she again returned to me. The thickening in the pelvis had disappeared, so that I was able to determine the exact condition. There was a dislocated ovary and tube. This was very tender. On the 13th of April, that is more than eleven months after the labor, I opened the abdomen with the assistance of Dr. Riggs, and in the presence of Dr. Foster and Dr. Hazzard. There were all the evidences of severe pelvic peritonitis. The right ovary and tube were prolapsed, the ovary itself was cystic, the pelvic peritoneum was covered with granulations. I removed the ovary and tube, and she recovered. She is now able to do almost all kinds of household work, and is entirely free from pain. I attribute the displacement of the ovary and tube, as well as the retroflexion and the pelvic peritonitis, to undue violence exercised by myself. I think the case is interesting and instructive, and at least calls for greater care in the use of this method of causing the expulsion of the placenta.

DR. BUCHANAN—I do not think it is clear that the Crede method is to blame for the result in the case. I think it would be very difficult to positively decide that it was violence done by the hand, and not infection, which was causative of the inflammation. According to the statement, there had been efforts at the removal of this placenta by attendant women before Dr. Munn arrived. He also made an examination, and although symptoms of trouble did not come until a week after the manipulations, I see no reason why this should militate against the probability of sepsis, especially as we have no account of the after-treatment, douches, etc. The related trouble cannot justly be ascribed to Crede's method. It is probable that this was a case of pelvic cellulitis or pelvic peritonitis from infection.

DR. BLUME.—Crede's method never produces septicæmia. Whenever the uterus is emptied by Crede's method, there may be left behind some shreds of membranes or parts of the placenta; these may become decomposed, and so cellulitis or local peritonitis may be the final result. This cannot be due to Crede's method. All cases of cellulitis, metritis and peritonitis, during the puerperium, are the result of infection. If the vagina is clean, the finger aseptic, or the instrument aseptic, there is all probability that by introducing the hands or instrument into the uterine cavity to remove parts of the placenta, sepsis will not follow. I wish to make one remark in regard to retroflexion of the womb. It is hard to understand how a womb as large, reaching to the umbilicus, can be retroflected by Crede's method. It might be possible about three or four days later. I believe it is not possible just after labor. There are many cases of Crede's method and its results reported, but as far as I know, none of retroflexion. The uterus is lying on the spine, and cannot be turned over.

DR. T. D. DAVIS.—I was struck by the same thought brought out by Dr. Buchanan. I do not think Crede's method is to blame for this trouble as much as is the doctor who fails to properly employ it. One of the advantages is that you retain complete control of the uterus by your hand. If you simply bear upon the fundus, you do not carry out Crede's method. Crede's method consists in grasping the fundus of the uterus with the fingers well placed around it, and holding it well within the grasp of the hand. The fingers and thumb grasp the uterus, and then by gentle pressure cause contractions of the uterus. No later than this morning I saw the placenta thrown into the bed by the powerful contraction of the uterus excited by that method. The conditions that followed in this case, whether they were septic and whether the pressure made by the doctor caused retroversion, the doctor is most qualified to determine.

DR. MUNN.—In stating the case, I said it was not an argument against the employment of Crede's method, but against the improper employment of it and the use of undue force by inexperienced persons. The point to which I wish to call attention is the possibility of doing damage when you attempt to employ Crede's method. As to whether this case was due to sepsis, it does not come under the classical description of sepsis. Fever should begin within five or six days. It was fully a week before this trouble began. I should attribute it to Crede's method, because I believe that the circulation of the uterus was more or less interfered with and the peritoneum was damaged, while the peritonitis was not developed until the seventh or eighth day. As to whether retroflexion of the uterus is possible or not, in this case it no doubt happened.

DR. MCMULLEN—I remember during last winter placing my ice cold hands over the uterus in a case of labor with hemorrhage. This was followed by a violent contraction, and the placenta was thrown clear out of the vagina. I tried this again a few times, and it produced the same effect. Cold is not sufficiently appreciated for this pupose. It is very effective. Dr. Munn is too severely just in blaming himself for the bad result of this case.

DR. RIGG.—The Crede method, I believe, is sometimes abused, particularly in the hands of inexperienced persons. Ten years ago I used the Crede method of expelling the placenta, not with the same discretion, however, that I should have employed it. Inversion followed. I replaced the uterus, and remained with the woman nearly all day to see whether anything would follow. There were hemorrhages until I got the uterus replaced. It is necessary to make light pressure when the uterus is up, when there is a contraction; and when there is no contraction relax the pressure. Simply stimulate the uterus when we have a contraction. I think with that precaution, we will seldom have any trouble from that method; I think it decidedly the best one we have.

Dr. Stewart exhibited an instrument for measuring the calibre of the male urethra, and read a paper on

THE DIAGNOSIS OF URETHRAL STRICTURE OF LARGE CALIBRE (SEE PAGE 411).

DR. MCCANN—I think this has certain advantages over urethrometers which I have examined. It seems of more value than any like instruments. All the others are exceedingly cumbersome, difficult to manipulate, and practically useless. The advantages in this are really the simplicity, and the fact that it lays before you a picture of the condition of the urethra. I am at a loss to understand how or why the urethrometer should be required for treatment of stricture which is twenty to forty millimetres in diameter. I find persons who have strictures in which number 16 English can be passed. I have been in the habit of telling such persons that they have no stricture, and I am satisfied this statement is true. The human urethra is not a tube like a gas pipe, it is not something which is invariable in its size and calibre; it varies with different individuals much as the size of the male organ varies. I heard a prominent professor, one of the leading professors in an eastern school, state that he had cut a man up to number forty, and the man came back again and stated he had another stricture, and he had felt it with his finger. Now, such treatment is malpractice, and malpractice of the grossest kind. The result is that men go through life dribbling their urine, being unable to propel a stream. These people are a profitable source of revenue to men who are unscrupulous. With such people I think the instrument invented by Dr. Stewart would probably be valuable; it would show them something tangible which they could gaze upon, and would satisfy them they had no stricture. But the stricture which will permit this instrument to pass will readily permit the passage of sounds of such size that the persistent use of them for a short time will cure the stricture.

